# Dynamic X-ray diffraction imaging of the ferroelectric response in bismuth ferrite

**DOI:** 10.1186/s40679-017-0044-3

**Published:** 2017-03-21

**Authors:** Nouamane Laanait, Wittawat Saenrang, Hua Zhou, Chang-Beom Eom, Zhan Zhang

**Affiliations:** 1 Center for Nanophase Materials Sciences, Oak Ridge National Laboratory, Oak Ridge, TN 37831 USA; 2 Institute for Functional Imaging of Materials, Oak Ridge National Laboratory, Oak Ridge, TN 37831 USA; 3 Department of Materials Sciences and Engineering, University of Wisconsin-Madison, Madison, WI 53706 USA; 4 X-ray Science Division, Argonne National Laboratory, Lemont, IL 60439 USA

## Abstract

X-ray diffraction imaging is rapidly emerging as a powerful technique by which one can capture the local structure of crystalline materials at the nano- and meso-scale. Here, we present investigations of the dynamic structure of epitaxial monodomain BiFeO_3_ thin-films using a novel full-field Bragg diffraction imaging modality. By taking advantage of the depth penetration of hard X-rays and their exquisite sensitivity to the atomic structure, we imaged in situ and in operando, the electric field-driven structural responses of buried BiFeO_3_ epitaxial thin-films in micro-capacitor devices, with sub-100 nm lateral resolution. These imaging investigations were carried out at acquisition frame rates that reached up to 20 Hz and data transfer rates of 40 MB/s, while accessing diffraction contrast that is sensitive to the entire three-dimensional unit cell configuration. We mined these large datasets for material responses by employing matrix decomposition techniques, such as independent component analysis. We found that this statistical approach allows the extraction of the salient physical properties of the ferroelectric response of the material, such as coercive fields and transient spatiotemporal modulations in their piezoelectric response, and also facilitates their decoupling from extrinsic sources that are instrument specific.

**Electronic supplementary material:**

The online version of this article (doi:10.1186/s40679-017-0044-3) contains supplementary material, which is available to authorized users.

## Background

Capturing the evolving structure of materials in functional devices under a host of varying thermodynamic potentials and environments, with nanoscale resolutions and in real-time, remains of one of the most actively pursued goals of structural imaging [1]. Out of the existing structural probes, X-ray diffraction-based microscopy enjoys a host of properties that are ideally suited to achieve the aforementioned goal: namely, the depth penetration of hard X-rays, their exquisite sensitivity to the atomic structure, and analytical power facilitated by their weak interactions with matter [2].

In contrast to well-developed structural probes such as dark field transmission electron microscopy [3], X-ray diffraction microscopy has been traditionally hampered by the availability of optics that can simultaneously operate in the hard X-ray regime and provide large numerical apertures for nanoscale resolution. Fortunately, the combination of continued advances in X-ray optics [4], the ever-increasing brightness of synchrotron sources [5], and sophisticated phase-retrieval algorithms has spurred a resurgence in X-ray diffraction-based imaging and has led to a diverse set of imaging modalities [6]. Of particular interest are those X-ray imaging modalities that place no restriction on the sample size nor its geometry and are of applicability to materials in thin-film form; one of the most technologically important class of materials. These modalities consist of: (i) nano- and micro-diffraction probes, whereby a focused beam is rastered across the sample to spatially map the diffracted intensity across a sample [7–9]; (ii) Bragg ptychography, where a real space image is reconstructed out of overlapping nano-diffraction patterns using phase-retrieval algorithms [10, 11]; (iii) full-field dark field X-ray microscopes which employ a combination of hard X-ray optics to form a real space image of the sample with diffraction contrast [12, 13].

Full-field dark field X-ray microscopes represent a novel extension of the widely used transmission X-ray microscopes, from an absorption-based contrast to diffraction contrast. These microscopes have been used to probe the static and dynamic structure of crystal surfaces [14, 15], reconstruct the three-dimensional crystal grain orientations in metals [13], and capture lattice rotations near misfit dislocation networks in complex oxide thin-films [16]. The highest spatial resolution that has been achieved in a full-field dark field X-ray microscope (sub-100 nm) [12] is coarser than that of both nano-diffraction probes (~few tens of nanometers) and Bragg ptychography (sub-10 nm), yet its temporal resolution is orders of magnitudes finer, with image acquisition times as short as few tens of milliseconds. As we show here, this combination of nanoscale resolution and high-frame rates enables full-field X-ray diffraction microscopes to probe the structural changes of crystalline materials in situ and in operando.

In this article, we demonstrate for the first time the capability to investigate the electric field-driven dynamic structural responses of buried monodomain bismuth ferrite epitaxial thin-films [17] in micro-capacitor devices at unprecedented spatiotemporal resolutions. By employing a full-field Bragg X-ray diffraction microscope (XDM), we demonstrate that a real space image with a field of view of ~15 × 15 μm^2^ and ~70 nm lateral spatial resolution is captured with an acquisition time as small as 50 ms. These imaging characteristics make XDM one of the most data-intensive, high-throughput synchrotron-based techniques, whose frame rates reach up to 20 Hz, and since each frame (i.e., a full image) contains 1024 × 1024 pixels (16-bit depth), its data transfer rates from the detector are as high as ~40 MB/s. By using a combination of diffraction contrast mechanisms, we probed the dynamically induced ferroelectric polarization and piezoelectric responses in bismuth ferrite as a function of electric field. To extract material responses from the large XDM datasets, composed of 10^2^–10^3^ images, we use matrix decomposition techniques such as independent component analysis. We found that this statistical-based approach allows the extraction of key material responses such as polarization coercive fields as well as transient spatiotemporal piezoelectric responses due to the onset of device fatigue. Furthermore, we find that matrix decomposition techniques also facilitating decoupling of the measured material responses from extrinsic sources present in the data that are instrument specific.

## Methods

### Sample and device preparation

**Fig. 1:**
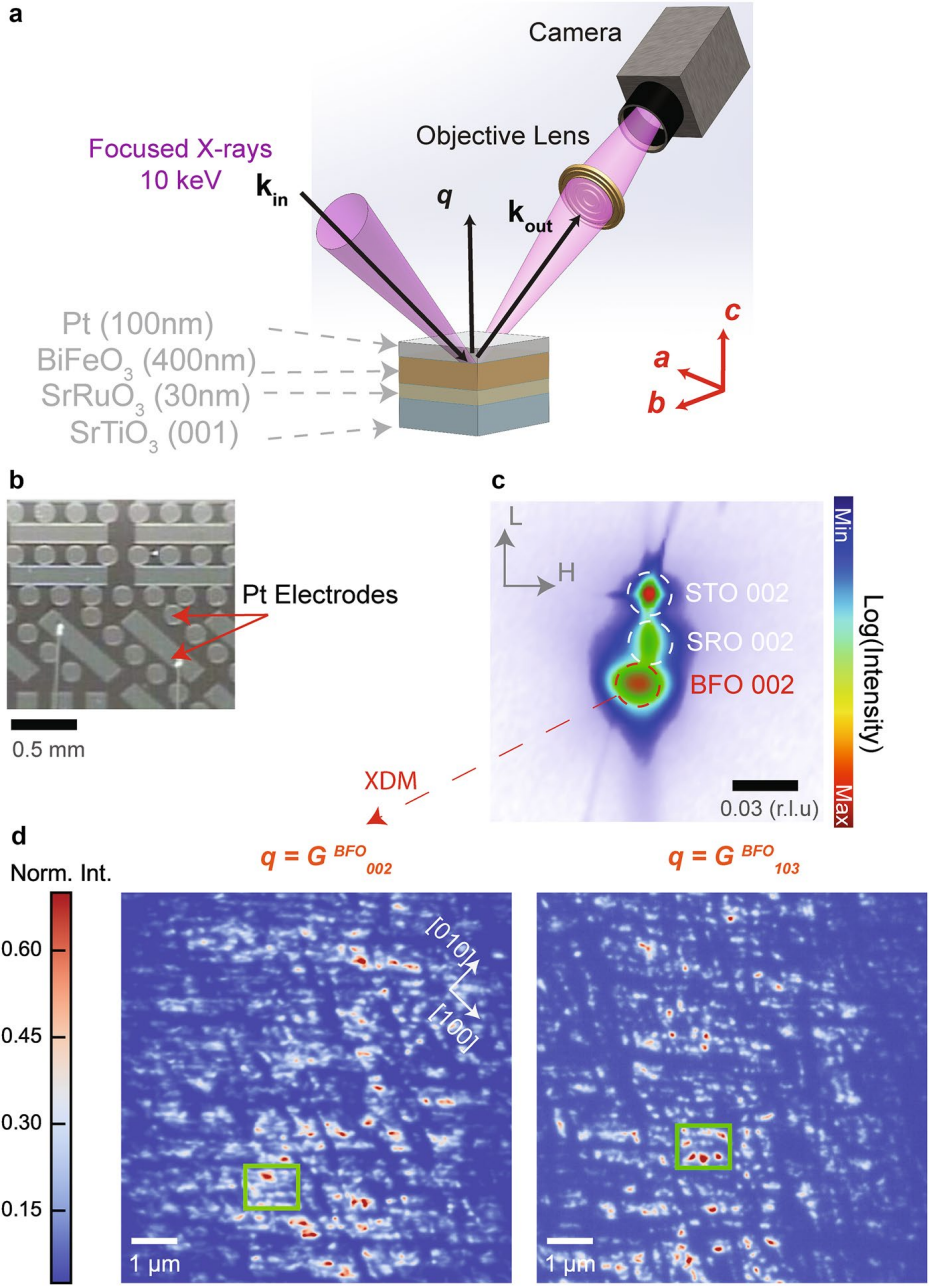
X-ray diffraction imaging of buried ferroelectric domains in a micro-capacitor configuration. **a** Schematic of the X-ray diffraction microscope (XDM), the scattering geometry, and characteristics of the sample (not drawn to scale). The imaging contrast of XDM is determined by the scattering vector, ***q*** = ***k***_out_ − ***k***_in_, where ***k*** ($|\varvec{k}| = 2\pi /\lambda$) is the wavevector of the X-ray wavefield ($\varvec{\lambda}\approx 1.2$ Å). A side view of the device indicates the thickness of the metallic electrodes [*top* Pt, *bottom* SrRuO_3_ (SRO)], and the ferroelectric thin-film BiFeO_3_ (BFO) that was grown on single crystal SrTiO_3_ (001). **b** Optical image of the device showing the Pt electrode patterning (top view). **c** Reciprocal space map (RSM) near STO 002 Bragg reflection. All reciprocal space coordinates are expressed in terms of SrTiO_3_ reciprocal lattice units (r.l.u). The BFO 002 Bragg reflection is offset in the HK-plane with respect to STO due to growth on a substrate with a high miscut angle (~4°, direction along STO [110]). **d** XDM images taken at different Bragg reflections of BFO (*left*: 002, *right*: 103). Spatial variations in the diffracted intensities are due to the presence of considerable lattice rotations (mosaicity) and epitaxial strain relaxation due to the large thickness of the film. *Boxes* in the images outline different mosaic blocks. The similarity in diffraction contrast between XDM images at** G**_002_ and** G**_103_ provide direct evidence for the mono-domain configuration of the ferroelectric thin-film (in agreement with RSM around STO 003, data not shown). The *color bar* indicates normalized diffracted intensity

Monodomain epitaxial BiFeO_3_ thin-films (400 nm) were grown by off-axis sputtering on a vicinal substrate of SrTiO_3_ (001) [18], with a metallic bottom electrode of SrRuO_3_ (30 nm) [19]. The miscut direction of the substrate is parallel to SrTiO_3_ [110] with the miscut angle of 4^o^. An array of micro-electrodes of Pt (~100 nm thick) were fabricated on the surface of the sample by photolithography (see Fig. 1a).

The samples were mounted on standard microchip carriers. Electrical grounding of the samples was performed by contacting the SrRuO_3_ bottom electrode, that is exposed from the side of the sample, with one of the terminals of the carrier using conductive silver paste. Wire-bonding with aluminum was used to connect different Pt electrode pads to the lead terminals of the chip carrier. In this micro-capacitor configuration, the BiFeO_3_ devices recorded resistances on the order of ~100 MΩ and large ferroelectric polarization values of ~70 μC cm^−2^. The electrical poling studies were performed using an arbitrary waveform generator (Tektronix) connected to a piezoelectric DC driver to provide well-defined voltage pulses.

### Reciprocal space mapping

Structural characterization of the samples was performed using synchrotron hard X-ray reciprocal space maps (RSM) at the Advanced Photon Source (33 BM-B) with an X-ray energy of 15.5 keV. Reciprocal space volumes near SrTiO_3_ (STO) 002 and 103 Bragg reflections were reconstructed from diffraction patterns by an area-based detector (Pilatus, Dectrix) acquired during angular scans of the Bragg angle at different angular inclinations of the sample with respect to the surface normal [20]. An HL-cut of the reciprocal space volume near STO 002 is shown in Fig. 1c. The large spread of the BiFeO_3_ (BFO) 002 reflection indicates the presence of substantial crystal mosaicity. These different mosaic block configurations are directly imaged in real space by X-ray diffraction microscopy (see below).

BiFeO_3_ is a well-known multiferroic, hosting both antiferromagnetism and ferroelectricity [21]. The crystal structure of BiFeO_3_ is rhombohedral with space group $R\bar{3}c$, where the polarization vector ***P*** points along ⟨111⟩ (crystal indexing based on a pseudo-cubic lattice is used throughout). By symmetry, ***P*** is eightfold degenerate (4 crystallographic directions ×2 polarity states) [22]. This degeneracy in ferroelectric polarization produces thin-films that contain different ferroelastic domain variants (up to 4). However, by growth on a vicinal substrate, all but one of the ferroelastic domain variants can be suppressed in favor of a film that is mono-domain and hosts a ferroelectric polarization vector with an in-plane component collinear with the miscut direction [110] and out-of-plane component that is parallel to [001]. In essence, by vicinal growth, ferroelectric ***P*** in our samples can only be twofold degenerate as it has been constrained to a single crystallographic direction, yet its polarity can be either up or down, switchable by an electric field. Note, that due to preferential screening of the depolarization field by the SrRuO_3_ (SRO) [23], the as-grown initial state of ***P*** in our samples has an out-of-plane component that is parallel to [001] and in-plane component that is parallel to $[\bar{1}\bar{1}0]$. We verified the mono-domain property of the BFO samples by RSM of BFO 103 and 113 reflections, showing no reflection peak splitting associated with a multi-domain configuration (Additional file 1: Figure S1) [24].

### X-ray diffraction microscopy

Structural imaging of the samples was performed at the Advanced Photon Source (XRIM instrument, 33ID-D). X-ray diffraction microscopy is a full-field imaging technique with an optical configuration that is composed of: (1) a condenser lens to illuminate the sample; (2) objective lens to form an image out of the Bragg diffracted wavefield. The condenser lens is composed of a pair of dynamically bendable Kirkpatrick–Baez mirrors that focus the X-ray beam (10 keV, 15 × 15 μm^2^) on the sample surface. The objective lens is a Fresnel zone plate (FZP) with 60 nm outermost zone width, that projects the image onto a sCMOS camera with a pixel size of 6.5 μm (Neo 5.5, Andor) that can be operated up to 100 frames per second (i.e., 100 Hz). The sCMOS camera is coupled to a scintillator for conversion from X-ray to optical, in addition to an oil-immersion lens (Nikon) for 20× optical magnification. The total magnification of XDM (~440×) is the product of FZP magnification (~22) and optical magnification, producing an effective pixel size of 15 nm on the sample and a field of view of ~15 × 15 μm^2^. The lateral resolution of XDM is ideally 60 nm and limited by the outermost zone width of the FZP. However, due to persistent mechanical vibrations in the instrument, the lateral resolution has been previously determined at ~70 nm. Additional experimental details on XDM have been reported elsewhere [12].

In this current configuration, XDM can acquire real space images of crystalline thin-films with Bragg diffraction intensities of thin-films on the order of 10^−5^ × *I*_o_ with signal-to-noise ratios of 10:1 in 1 s, where *I*_o_ ∼10^12^ photons/s is the incident flux on the sample. Due to the scaling of thin-film diffraction as *N* ^2^, where *N* is the number of coherently scattering thin-film layers, thicker films inherently lead to stronger signals and images with field of view and lateral resolution specified above can be acquired in 10s of milliseconds (see below).

The contrast in XDM images is determined entirely by the scattering vector $\varvec{q}_{{\varvec{HKL}}}$, where *HKL* are the Miller indices (see Fig. 1a). Representative images of BFO acquired at the 002 and 103 Bragg reflections are shown in Fig. 1d, with $\varvec{q} = \varvec{G}_{002}$ and $\varvec{q} = \varvec{G}_{103}$, respectively. For every image pixel (*x,y*) is associated a diffracted intensity, *I*_***q***_ (*x,y*), from a region of the film whose real space location on the sample surface is (*x,y*). Due to the depth penetration of 10-keV X-rays (~microns), an XDM image contains information that is sensitive to the full three-dimensional spatial distribution of (HKL) planes, with a lateral spatial resolution that is on the order of ~70 nm (5 × pixels) but with imaging contrast that is diffraction limited (resolution ~ *d*_*HKL*_ */*2). Both XDM images in Fig. 1d show the presence of mosaic blocks on the order of a micron (outlined in Fig. 1d). The intensity variations across the images originate from (002) and (103) lattice plane rotations introduced by various epitaxial strain relaxation mechanisms, which moves their scattering out of the Bragg condition. The spatial variations in these lattice rotations from one mosaic block to another can be directly extracted from XDM images [16]. Furthermore, note that the similarity in XDM image contrast between the 002 and 103 reflections is further confirmation that the BFO samples are indeed in a mono-domain configuration, since splitting of the 103 reflection by multiple domain variants would substantially modify the XDM contrast, leading to the appearance in XDM images of real space ferroelastic domain patterns along preferred crystallographic orientations, to satisfy mechanical compatibility conditions of BiFeO_3_ [25] (e.g., see [12] for XDM imaging of ferroelastic domain walls).

## Results

### Imaging of the out-of-plane ferroelectric response

**Fig. 2:**
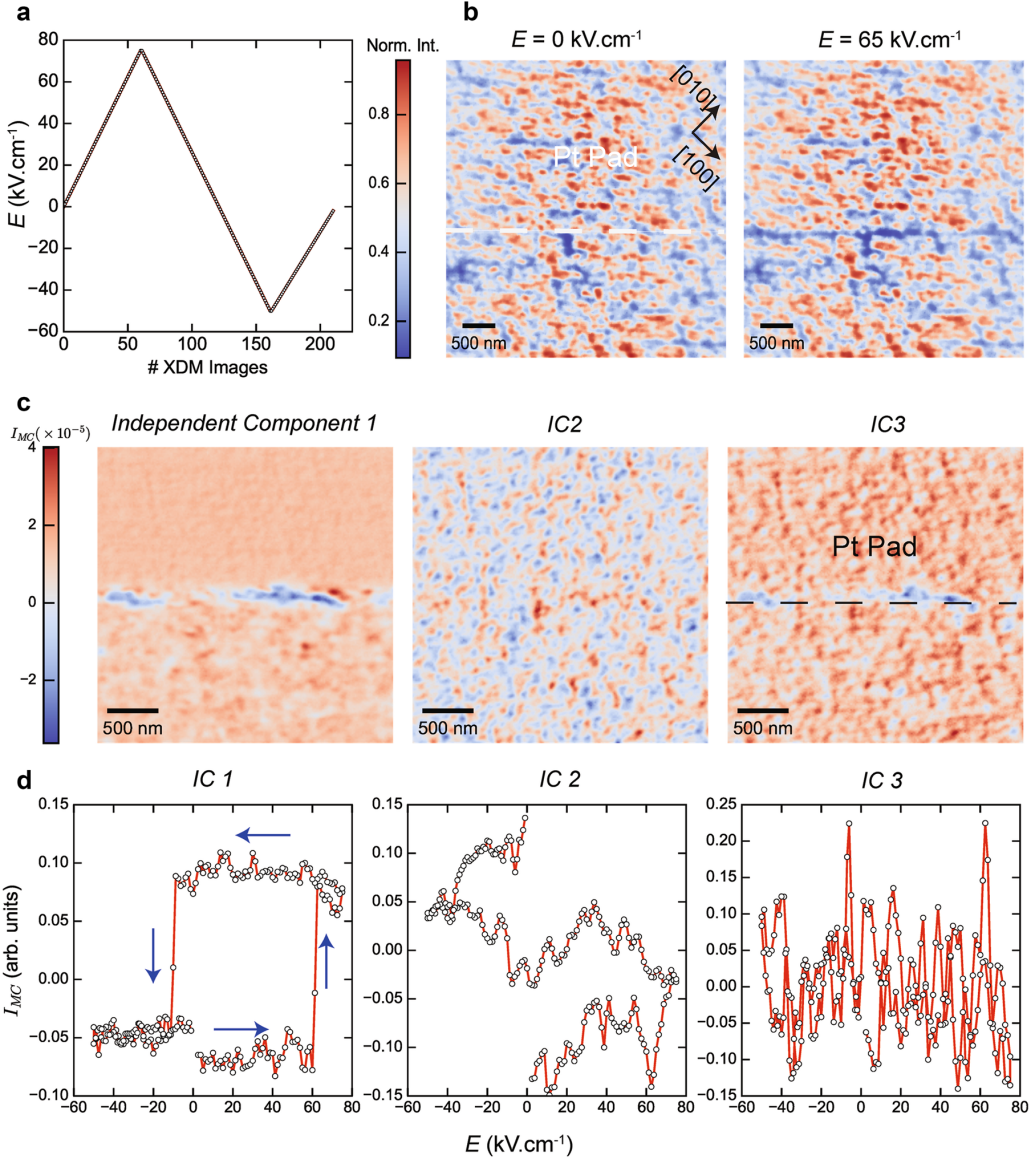
Imaging the ferroelectric switching of BiFeO_3_. **a** The poling cycle applied in the presented XDM dataset, where *E* is the electric field between the Pt and SRO electrodes and is oriented along [001]. At each point an electric pulse lasting 0.5 s is applied, followed by acquisition of an XDM image (acquisition time 0.2 s) with $\varvec{q}_{0,0,2 - \delta }$, where *δ* ≈ 10^−1^. **b** Representative XDM images taken at different values of the electric field cycle in **a**. The *dashed line* in the *right panel* indicates the edge of the Pt electrodes. After poling the BFO film underneath the Pt electrode, the domain-wall between two regions of the film with opposite out-of-plane polarization vectors produces amplitude contrast (negative) in diffraction imaging. The entire collection of XDM images acquired during the electric field cycle (~200 images) are unmixed using independent component analysis (ICA), assuming three independent components or sources. In **c** the XDM collection is projected onto these three different basis vectors and reshaped into images, while **d** shows the corresponding evolution of these independent components (IC) as a function of electric field. The landmark hysteresis polarization loop of BFO is obtained directly from IC1 and its spatial map in **c** shows the domain wall between the two opposite polarization states. The *arrows* in **d** indicate the direction of the poling cycle. The other two ICA components IC2, three are mostly closely associated with extrinsic instrumental factors that enter the data such as X-ray beam intensity fluctuations or drift, given that their evolution during the poling cycle is not physically relatable to ferroelectric behavior. *I*_*MC*_ represents the mean-centered intensity, whereby the mean intensity in each image in the original data is subtracted from the XDM collection before applying ICA

Taking advantage of the depth penetration of hard X-rays, we acquired images of the changes in the local structure of BFO buried underneath the 100 nm Pt pads during electrical poling. The electric field ***E*** was applied between Pt and SRO and is oriented along the *c*-axis of the sample. A full poling cycle was performed (Fig. 2a), during which an XDM image was acquired at each value of ***E***.

Ferroelectric materials such as BFO also exhibit a converse piezoelectric effect, whereby an applied electric field leads to expansion/contraction of the material [26]. This change in the material causes a shift in the scattering angle (2*θ*) at a Bragg reflection (e.g., 002) and is one method by which to achieve imaging contrast (see below). Here, we suppressed XDM contrast sensitivity to the converse piezoelectric by offsetting the scattering vector to $\varvec{q}_{0,0,2 - \delta }$, with *δ* ≈ 10^−1^. XDM images taken at this scattering condition under zero field and 65 kV cm^−1^ show the formation of a domain wall once the polarization of the BFO underneath the Pt pad is switched. The presence of (dark) image diffraction contrast from a ferroelectric domain wall can be qualitatively understood using a well-known result from Fourier optics [27]; any pure phase object (such as up and down polarization states) that is imaged by a microscope with a limited transfer function, produces an intensity dip precisely at the location of the phase change (i.e., location where the polarization direction changes). The offset from the Bragg condition, $\delta \approx 10^{ - 1}$, was empirically tuned to maximize intensity contrast for the domain wall, but could be determined, in principle, from a full analysis of diffraction contrast as well as optical responses of XDM.

To extract the fundamental properties of the system’s ferroelectric response from the XDM images, the field-driven changes in image contrast must be analyzed. Note that over the course of a single poling cycle, more than 200 images were acquired at increments of 1.25 kV cm^−1^; such a fine resolution is needed given the abrupt change in polarization. To mine such large data sets in an unsupervised and statistically rigorous manner we used independent component analysis (ICA) [28]. ICA is a matrix decomposition technique that is commonly used for blind source separation of different statistically (linearly) independent source signals present in a dataset [29]. The main assumption of ICA, namely, linear independence between sources is of wide applicability and has been successful in extracting artifacts from the imaging of neuronal activities [30], as well as extracting hidden structure from financial data [31].

In applying ICA to our data, we note that material responses due to an electric field should exhibit hysteresis, while extrinsic sources not associated with ferroelectricity will not display hysteretic behavior as a function of an electric field. As such, the main assumption of statistical independence of ICA, should allow blind source separation between these different signal classes, without any a priori information or model-dependent analysis.

We denote the field-dependent XDM images by $\varvec{X}\left( {\varvec{r},\varvec{E}} \right),$ then ICA performs the following decomposition:

1 $$\varvec{X}(\varvec{r},\varvec{E}) = \mathop \sum \limits_{i = 1}^{N} \varvec{A}_{i} (\varvec{r})S_{i} (\varvec{E}),$$

where ***r*** = (*x,y*) are the sample spatial positions, ***A***_***i***_ are column vectors of the mixing matrix ***A***, and *S*_***i***_ are the independent sources or components (IC). Note that with each source *S*_***i***_ is associated an image ***A***_***i***_, which can be thought of as a projection of the full data set ***X*** onto the basis vector of the pure source *S*_***i***_. Therefore, ***A***_***i***_ provides a representative image that encapsulates the main spatial features or information associated with source *i*.

The results from the independent component analysis on the field-dependent XDM data are shown in Fig. 2c, d, which represent the spatial projections of the source [i.e., $\varvec{A}_{i} \left( \varvec{r} \right)$ in Eq. 1] and the field-evolution of the sources [i.e., $S_{i} (\varvec{E})$], respectively. The first IC projection shows the domain wall that is formed as the polarization is switched while its field dependence shows the classic polarization hysteresis loop. Interestingly, the hysteresis loop is not centered around the zero-field condition, indicating that the Pt/BFO and SRO/BFO interfaces contains trapped charges that preferentially screen the polarization. Screening of ferroelectric polarization by charges and associated modulations in the polarization hysteresis curve due to the Schottky barrier formed at the metal/semiconductor (ferroelectric) junction is a notorious problem in thin-film ferroelectrics, and is one of the most actively investigated topics in thin-film ferroics [32].

The remaining components, IC2(3) are associated with sources whose field-dependence is not directly relatable to polarization switching and are likely artifacts present in the raw data such as X-ray beam intensity fluctuations or sample drift. Note that analysis of the XDM data by using an explicit model would have been heavily influenced by IC2 and IC3. The advantage of ICA resides in the un-mixing of statistically independent sources in an automated, and statistically unbiased fashion. In addition, ICA can also be viewed as a dimensionality reduction technique, where each data point in plots of the independent components was originally an XDM image with 1024 × 1024 pixels.

### Imaging of the in-plane ferroelectric response

**Fig. 3:**
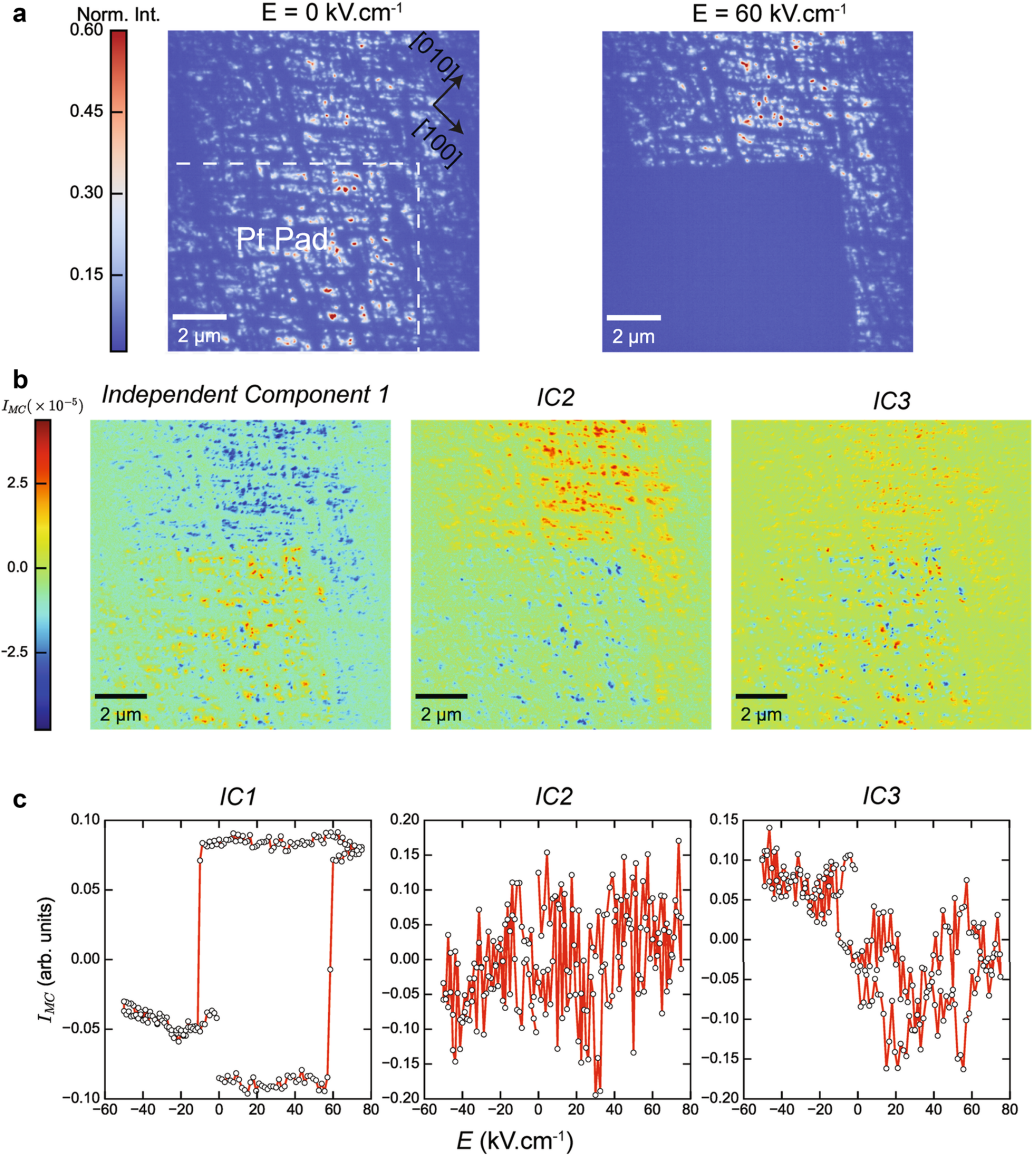
Imaging the in-plane piezoelectric response of BiFeO_3_. XDM images were acquired with $\varvec{q} = \varvec{G}_{103}^{\mathbf{BFO}}$, during the poling cycle in Fig. 2a. Representative images taken at different values of the electric field during the cycle are shown in **a**. The *dashed line* represents the boundary of the Pt electrode. At *E* = 60 kV cm^−1^, the 103 Bragg planes of BFO underneath the electrode are rotated as a consequence of ferroelastic switching by shear strains, and consequently they no longer satisfy the Bragg condition at $\varvec{q} = \varvec{G}_{103}^{\mathbf{BFO}}$ and appear in the XDM image with dark contrast (*left panel*). **b** Independent component analysis of the 103 XDM collection as a function of poling cycle, showing both the projections of the collection along the three basis vectors (*top*), and the evolution of the unmixed components as a function of electric fields (*bottom*). **c** Similarly, to the 002 XDM poling cycle, ICA component 1 shows the expected hysteresis as a function of electric field, with the remaining components encoding non-ferroelectric, extrinsic sources in the data

We repeated the same poling studies shown earlier, but instead acquired XDM images at a different scattering condition $\varvec{q} = \varvec{G}_{103}^{\mathbf{BFO}}$. At this scattering condition, XDM imaging contrast is sensitive to both in-plane and out-plane changes in the structure of BFO. Given that the BFO film is strain relaxed and its polarization vector is along $[\bar{1}\bar{1}1]$, we expect that the applied electric field (along [001]) will couple not only to piezoelectric coefficients along the *c*-axis (i.e., *d*_*33,*_ *d*_*ij*_ is the piezoelectric strain tensor), but also to shear strains (e.g., *d*_13_) that we can image by XDM. Remarkably, at a field of 65 kV cm^−1^ all contrast from BFO domains underneath the Pt pad disappears entirely (Fig. 3a). Despite the presence of significant lattice rotations by epitaxial strain relaxation near the boundaries of the mosaic blocks, the polarization response of the sample is found to be homogeneous over distances that are an order of magnitude larger than the size of a mosaic block. The field-dependent XDM data are analyzed by ICA with Fig. 3b, c displaying the spatial maps associated with the independent components and the latter’s dependence on the electric field, respectively. Similarly to the independent sources found in XDM data acquired at $\varvec{q}_{002}$, we find that one of the components produces the hysteresis loop (IC1) with identical physical properties such as the ferroelectric coercive fields (65, −15 kV cm^−1^) as well as asymmetry with respect to the zero-field condition due to polarization screening that was discussed earlier (see Fig. 2d). For completeness, we note that in addition to changes in *d*-_*13*_, rotations of (103) lattice planes of BFO (with respect to [110]) may also occur under an applied electric field, leading to *ferroelastic* switching. The changes in XDM imaging contrast due to these different material responses is indistinguishable, if measurements are taken at a single scattering vector $\varvec{q}$, as was performed here. However, by independent reciprocal space measurements of the BFO 113 Bragg reflection as a function of electric field (see Additional file 1: Figure S2), we are able to determine that the observed changes in XDM contrast are mainly due to changes in *d*_*13*_.

### Dynamic imaging of the piezoelectric response

**Fig. 4:**
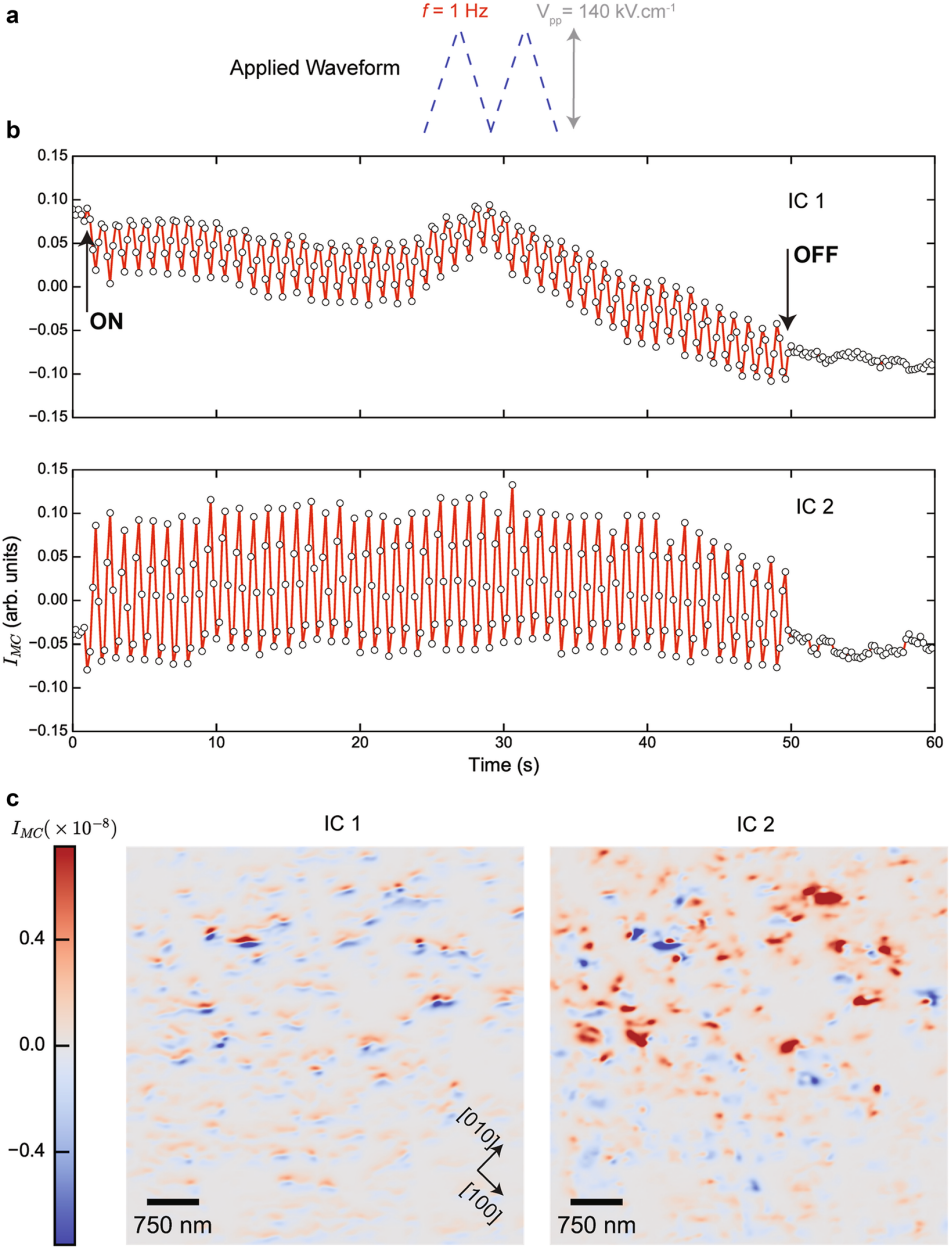
Dynamic imaging of the poling response. **a** An electric field modulated with a triangular waveform is applied across the BFO film. Simultaneously, XDM images were acquired at $\varvec{q} = \varvec{G}_{002}^{\mathbf{BFO}}$ at a frame rate of 5 Hz. By decomposition of the XDM collection with ICA, the correct characteristics of the waveform can be extracted directly from the XDM data without prior knowledge of the waveform characteristics in **a**. Note that each point in the time-series corresponds to a full XDM image. We found two components in the data, both showing the same intensity modulation frequency, but with different response amplitudes (**b**). The spatial maps in **c** (*left*) show the spatial distributions of domains whose response amplitudes are both weaker and vary with time (corresponding to IC1), while the *right panel* shows the spatial distributions of BFO domains that have a constant amplitude response that follows the applied waveform in **a** as given by IC2 in **b**

In addition to the static imaging presented so far, we dynamically imaged the field-driven structural responses of the film during the application of multiple triangular waveform cycles with varying frequencies. In Fig. 4a, an example of the applied waveform is shown while XDM images at the BFO 002 Bragg reflection were acquired at a frame rate of 5 Hz. This results in a time-dependent XDM set, $\varvec{X}(\varvec{r},t)$. Some of the information that can be extracted from this data is the nanoscale spatial inhomogeneity in the response of BFO domains, as well as temporal variations in their response, for instance due to device fatigue or influence of localized defects near domain boundaries. As such, all of the presented dynamic data were taken on a pristine micro-capacitor and the total number of poling cycles applied to the device was recorded. We extract these information channels by independent component analysis in the time-domain as follows:

$$\varvec{X}(\varvec{r},t) = \mathop \sum \limits_{i = 1}^{N} \varvec{A}_{i} (\varvec{r})S_{i} (t),$$

where the independent components *S*_*i*_(*t*) now acquire time-dependence, and associated with each is a spatial feature map $A_{i} (\varvec{r})$. By decomposing $\varvec{X}(\varvec{r},t)$ into two pure sources, we extracted the main characteristics of the applied waveform (i.e., frequency, slope) without a priori knowledge of the applied waveform. The observed oscillations in the intensity correspond to shifts in the scattering angle 2*θ* due to the converse piezoelectric effect. We found two types of time-dependent piezoresponses in the BFO film, with IC2 showing a constant amplitude as a function of time that closely tracks the alternating field, while IC1 displays a response with an amplitude that is modulated over longer times (~10 s) but over shorter time scales displays the same frequency as IC1 and the applied waveform. Interestingly, the spatial feature map that is associated with IC1 shows that BFO domains whose piezoresponse is modulated with a longer period are largely oriented parallel to the miscut direction [110]. These modulations could be indicative of the presence of different strain gradients or local defects in BFO near film domain boundaries oriented along the miscut direction which would surely affect the piezoelectric response of the system.

**Fig. 5:**
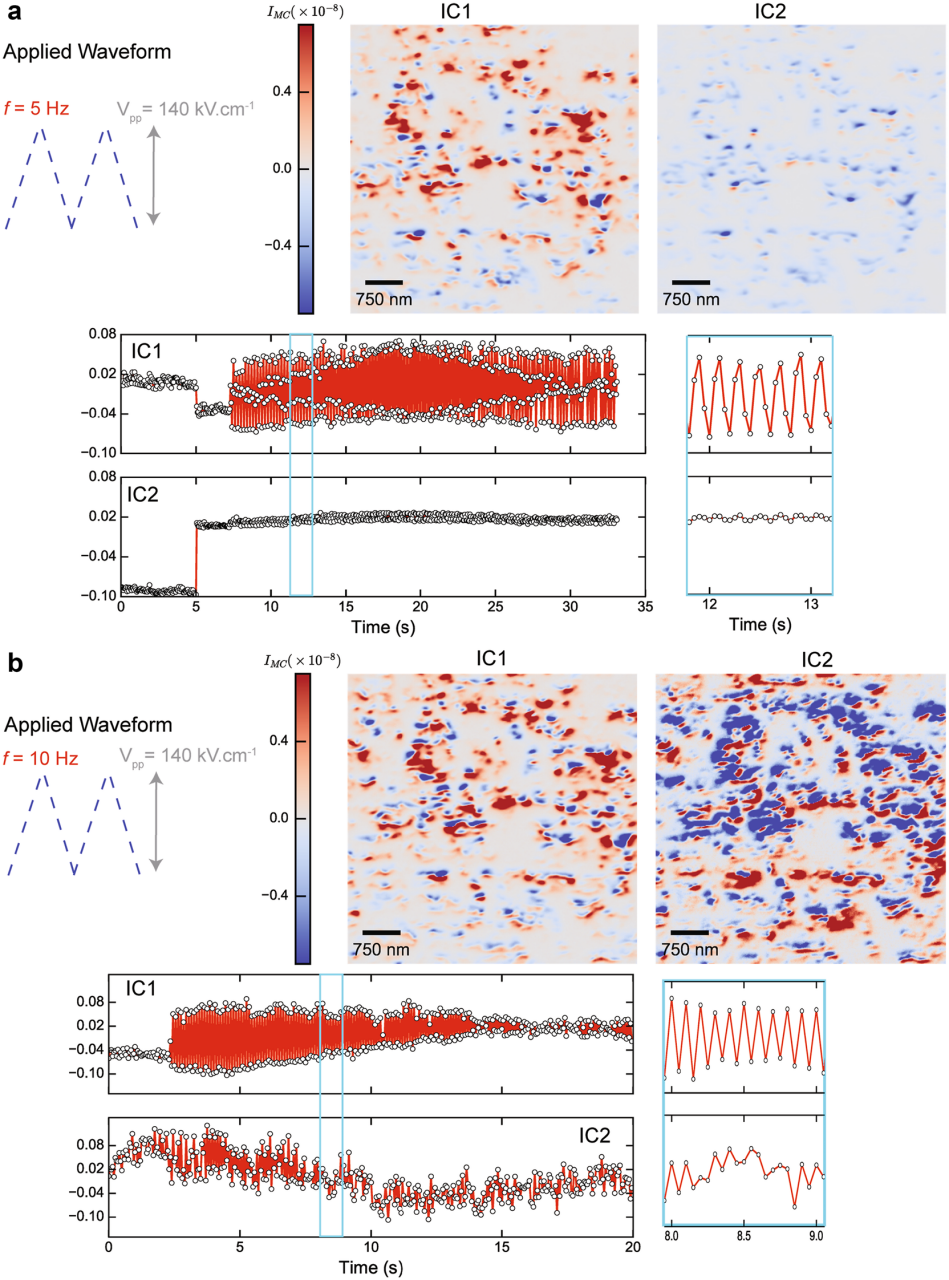
Detecting transient responses by dynamic X-ray imaging. Triangular electric field waveforms were applied to the BFO films at frequencies of 5 Hz (**a**) and 10 Hz (**b**). Simultaneously, XDM images were acquired at frame rates of 20 Hz, respectively. Note that each point in the time-series corresponds to a raw XDM image. By un-mixing the signals found in XDM by independent component analysis, we extract both the spatial distributions of BFO domains that respond synchronously to this waveform (**a**), but also find the onset of time-dependent reduced piezoelectric response due to polarization fatigue. In **b** independent component 2 shows larger intensity fluctuations than IC2 in **a**, indicating the onset of transient piezoelectric response. The *rectangular boxes* in the plots of the independent components (*left*) indicate the time intervals from which the close-up views are shown (*right panels* in **a**, **b**)

Following the same procedure as above, we applied waveforms with frequencies of 5 and 10 Hz, while capturing images at frame rates of 20 Hz (see Fig. 5a, b). In the first case (5 Hz), no transient dynamics were detected; the piezoelectric response of the film reflects one-to-one the field modulation (IC1). Note that this is in contrast to the behavior observed in the pristine state (Fig. 4b), and indicates that the modulated piezoelectric response found in Fig. 4b (top) is likely due to charge defects and not strain gradients due to this irreversible behavior. The second independent component describes the behavior of regions of the film that are on the order of ~100 nm that show no response to the electric field (IC2).

After poling the device continuously with nearly 3 × 10^3^ cycles, we applied a 10-Hz waveform (Fig. 5b). During the first 5 s of poling the film, the measured piezoelectric response was synchronous with the waveform (as shown in the close-up). Progressively, however, the total amplitude of the response was observed to diminish and became barely detectable at *t* > 12 s. Note that the spatial feature map of IC1 closely resembles that of IC1 from the 5-Hz waveform, indicating that no changes in the spatial configuration of BFO domains with strong piezoelectric response has occurred. Moreover, we find that much larger fluctuations in IC2 are present, as can be clearly seen by comparing their time series. Furthermore, the spatial features map (IC2 in Fig. 5b) now displays a much larger density of BFO domains whose response fluctuates substantially and in a transient fashion. These observations point to the onset of polarization fatigue, and was immediately confirmed by electrical measurements on this device, showing an increase of three orders of magnitude in resistance from ~100 MΩ to ~100 kΩ.

## Discussion

Through the judicious application of model-independent matrix decomposition techniques, such as independent component analysis, we were able to effectively mine the large datasets produced by XDM, thereby extracting many of the static physical characteristics of ferroelectric phenomena in bismuth ferrite thin-films such as the coercive fields, and to infer the presence of preferential electrostatic screening at the Pt/BiFeO_3_ interface. In addition, by applying ICA to time-dependent XDM data, we detected time-dependent modulated behavior in the piezoelectric response in the pristine state that is likely caused by the preferential local concentration of vacancies near domain boundaries oriented parallel to the miscut crystallographic orientation. After repeated polarization cycling, we were also able to detect the onset of transient piezoelectric switching that is indicative of the onset of polarization fatigue. In each case, spatially resolved maps of the changes in the structure of BFO, associated with each observed response, were also obtained, facilitating not only the interpretation of the observed phenomena but also served as a meaningful method by which to visualize the main spatial information that is essentially spread out over ~10^2^–10^3^ images, each with 1024 × 1024 pixels.

The main advantage of the presented analysis is clearly its model-independent nature; no assumptions were made regarding the presence of: (i) hysteretic signals in the static imaging (Figs. 2, 3); (ii) oscillatory piezoelectric responses during the dynamic imaging (Figs. 4, 5). Note also that in each case of dynamic imaging, through the use of ICA we were able to extract the applied waveforms without implicit knowledge of the latter. More importantly, the extracted material response signals were discerned from spurious sources contained in the data, simply by their (linear) statistical independence, leading to the un-mixing of the two classes of signals and essentially producing material responses that are artifact-free. The latter is an important attribute of this decomposition technique, and one can therefore consider its application as an exploratory data mining technique in high-throughput X-ray imaging, followed by more physics-based model-dependent analysis. Note that for nearly all matrix decomposition techniques their robustness and applicability can only be measured by their empirical performance by subsequent comparisons to physics-based modeling to establish their utility in a particular class of problems.

The decorrelation of extrinsic sources from intrinsic signals present in the large datasets produced by full-field X-ray diffraction microscopy is an indispensable component in further development of X-ray diffraction-based imaging techniques and their application to materials physics. By their very nature, synchrotron sources are noisy environments, and consequently an X-ray microscope that extends over tens of meters (from monochromator to detector) in this environment will invariably produce datasets that contain extrinsic signals. These signals have been consistently identified in the presented results (e.g., Fig. 2d) and can consist of increased variations in the spatial distributions of the X-ray beam or mechanical instabilities. It would be interesting in future work to incorporate matrix decomposition models that explicitly incorporate different noise models (e.g., noisy ICA [28]) for a more robust un-mixing of material responses from instrumental responses.

## Conclusion

We have presented full-field Bragg diffraction imaging studies of the field-driven responses in epitaxial thin-films at unprecedented spatiotemporal resolutions. We also demonstrated how model-independent analysis techniques facilitate mining these immense datasets for the salient material responses and their spatiotemporal evolution. Future developments in this type of microscopy and associated data-intensive algorithms to mine its large datasets, promise to enable access to a host of field-driven structural responses in materials at the nanoscale and in real-time.

## Additional file

**Additional file 1: Figure S1.** Reciprocal space mapping of BiFeO_3_/SrRuO_3_/SrTiO_3_ at the 103 and 113 Bragg reflection in the pristine state (i.e., zero applied electric fields). **Figure S2.** Reciprocal space mapping of BiFeO_3_/SrRuO_3_/SrTiO_3_ at the 113 Bragg reflection as a function of applied electric field.

## Abbreviations

XDM: X-ray diffraction microscopy
ICA: independent component analysis
STO: SrTiO3
BFO: BiFeO_3_
SRO: SrRuO_3_
