# Supplementary material for: Dynamic X-ray diffraction imaging of the ferroelectric response in bismuth ferrite
Source: Adv Struct Chem Imaging. 2017 Mar 21;3(1):11. doi: 10.1186/s40679-017-0044-3 (PMC5477694; doi:10.1186/s40679-017-0044-3)
Supplement: Supplementary file 1 — Additional file 1: Figure S1. Reciprocal space mapping of BiFeO3/SrRuO3/SrTiO3 at the 103 and 113 Bragg reflection in the pristine state (i.e., zero applied electric fields). Figure S2. Reciprocal space mapping of BiFeO3/SrRuO3/SrTiO3 at the 113 Bragg reflection as a function of applied electric field. [file 40679_2017_44_MOESM1_ESM.pdf]

Supplementary Information: Dynamic X-ray Diffraction Imaging of the Ferroelectric Response in Bismuth Ferrite

Nouamane Laanait[1,2*], Wittawat Saenrang[3], Hua Zhou[4], Chang-Beom Eom[3], Zhan Zhang[4]

[1] Center for Nanophase Materials Sciences, Oak Ridge National Laboratory, Oak Ridge, TN 37831, USA

[2] Institute for Functional Imaging of Materials, Oak Ridge National Laboratory, Oak Ridge, TN 37831, USA

[3] Department of Materials Sciences and Engineering, University of Wisconsin-Madison, Madison, WI 53706, USA

[4] X-ray Science Division, Argonne National Laboratory, Lemont, IL 60639, USA

*Correspondence: laanaitn@ornl.gov

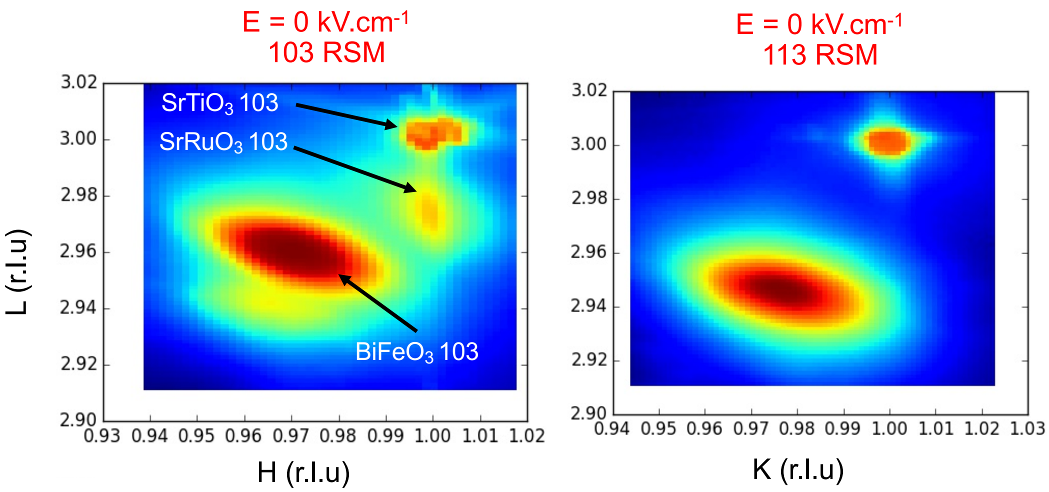

Figure S1. **Reciprocal space mapping of $BiFeO_3/SrRuO_3/SrTiO_3$ at the 103 and 113 Bragg reflection in the pristine state (i.e. zero applied electric fields)**. The absence of BFO peak splitting provides due to coherent twinning by ferroelastic structural variants, gives direct evidence of the mono-domain state of our samples. The reciprocal space maps are indexed using reciprocal lattice units (H, K, L) of $SrTiO_3$ (001).

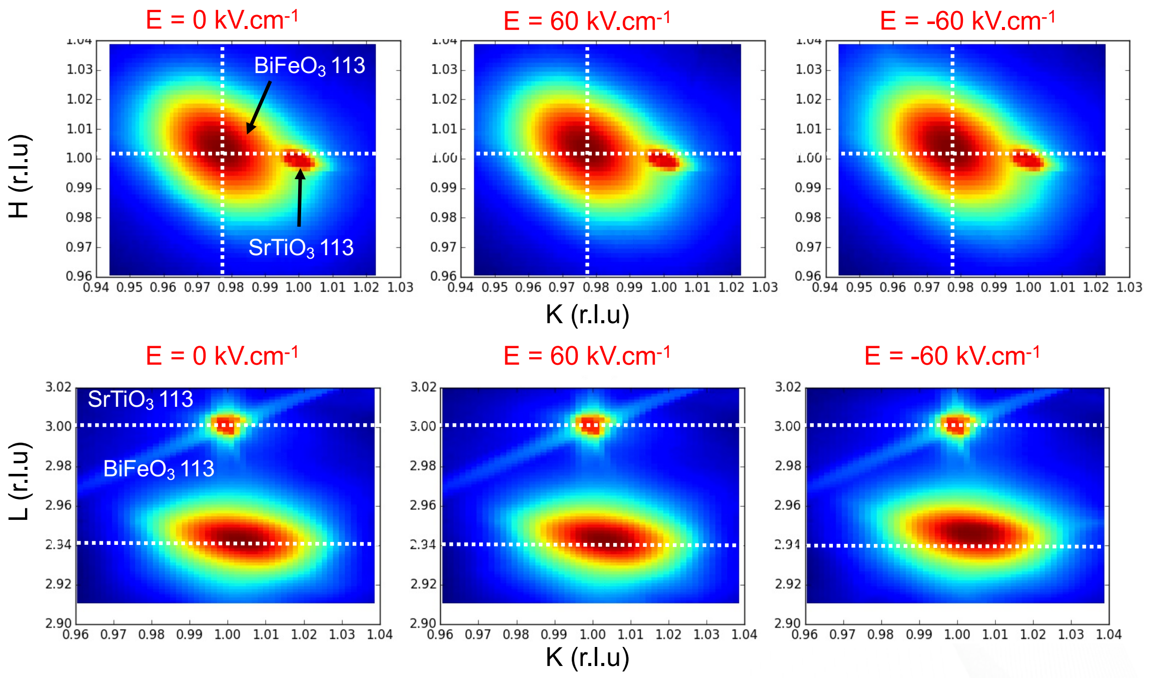

Figure S2. **Reciprocal space mapping of $BiFeO_3/SrRuO_3/SrTiO_3$ at the 113 Bragg reflection as a function of applied electric field**. Lines across the figures panels are a guide to the eye and were drawn at identical HKL positions in each reciprocal space map. By taking different cuts (HK, KL), we confirmed that the changes in the crystal structure of BFO due to electric field is dominated by changes in lattice parameters (shift in L of bottom panel) as opposed to lattice rotations with respect to [110], which would have resulted in shifting the reflection in the HK plane (top panel). The reciprocal space maps are indexed using reciprocal lattice units (H, K, L) of $SrTiO_3$ (001).
